# Matrix Metalloproteinase-9 and Tissue Inhibitor of Matrix Metalloproteinase-1 in Sepsis after Major Abdominal Surgery

**DOI:** 10.1155/2018/5064684

**Published:** 2018-05-16

**Authors:** Suzana Bojic, Jelena Kotur-Stevuljevic, Aleksandra Aleksic, Jasna Gacic, Lidija Memon, Sanja Simic-Ogrizovic

**Affiliations:** ^1^Department of Anaesthesiology and Intensive Care, CHC Bezanijska Kosa, 11000 Belgrade, Serbia; ^2^Faculty of Pharmacy, University of Belgrade, 11000 Belgrade, Serbia; ^3^Department of Surgery, CHC Bezanijska Kosa, 11000 Belgrade, Serbia; ^4^Clinical Chemistry Laboratory, CHC Bezanijska Kosa, 11000 Belgrade, Serbia; ^5^School of Medicine, University of Belgrade, 11000 Belgrade, Serbia

## Abstract

**Background:**

The role of matrix metalloproteinase-9 (MMP-9) and tissue inhibitor of matrix metalloproteinase-1 (TIMP-1) in sepsis after major abdominal surgery and sepsis-associated organ dysfunction is unexplored.

**Materials and Methods:**

Fifty-three patients with sepsis after major abdominal surgery were compared to 50 operated and 50 nonoperated controls. MMP-9, TIMP-1, biomarkers of inflammation, kidney and liver injury, coagulation, and metabolic disorders were measured daily during 96 h following diagnosis of sepsis and once in controls. MMP-9/TIMP-1 ratios and disease severity scores were calculated. Use of vasopressors/inotropes, mechanical ventilation, and survival were recorded.

**Results:**

Septic patients had lower MMP-9 and MMP-9/TIMP-1 ratios but higher TIMP-1 levels compared to controls. AUC-ROC for diagnosis of sepsis was 0.940 and 0.854 for TIMP-1 and 0.924 and 0.788 for MMP-9/TIMP-1 ratio (sepsis versus nonoperated and sepsis versus operated controls, resp.). Lower MMP-9 and MMP-9/TIMP-1 ratio and higher TIMP-1 levels were associated with shorter survival. MMP-9, TIMP-1, and MMP-9/TIMP-1 ratio correlated with biomarkers of inflammation, kidney and liver injury, coagulation, metabolic disorders, and disease severity scores. Use of vasopressors/inotropes was associated with higher TIMP-1 levels.

**Conclusions:**

MMP-9, TIMP-1, and MMP-9/TIMP ratio were good diagnostic or prognostic biomarkers of sepsis after major abdominal surgery and were linked to sepsis-associated organ dysfunction.

## 1. Introduction

Sepsis, a systemic inflammation caused by infection, is currently the leading cause of death in surgical intensive care units (SICUs) [1] with a mortality rate approaching 65% [2]. An overwhelming inflammatory response, the hallmark of this condition, could lead to the dysfunction of virtually every major organ or tissue [3].

Matrix metalloproteinase-9 (MMP-9), a zinc-dependent proteinase, is released by various inflammatory cells, predominantly neutrophils and macrophages. Together with its endogenous inhibitor, tissue inhibitor of metalloproteinase-1 (TIMP-1), it regulates numerous signaling pathways of pivotal importance in inflammation [4–6].

The association of MMP-9 and TIMP-1 with severity and outcome of sepsis has already been recognised [7–15]. However, these biomarkers were not explored in surgical sepsis which is considered significantly different from sepsis in medical patients [16] or in major abdominal surgery per se. Also, aside from acute kidney injury (AKI) [17], the association of MMP-9 and TIMP-1 with the clinical manifestations of sepsis-associated organ dysfunction has not yet been investigated despite numerous animal models of sepsis that clearly demonstrate elevated expression of these biomarkers in the kidney [18], lung [18–21], liver [18, 21], brain [22], heart [23], and endothelium [24].

The objectives of this study were to investigate, for the first time, MMP-9, TIMP-1, and the MMP-9/TIMP-1 ratio as diagnostic and prognostic biomarkers of sepsis after major abdominal surgery as well as the potential relationships between these biomarkers and sepsis-associated organ dysfunction.

## 2. Materials and Methods

### 2.1. Design and Patients

This prospective, observational study included 153 consecutive consenting adult patients admitted to the University Hospital Department of Surgery and SICU. The study has been approved by the institution's Ethics Committee. Written consents were obtained from patients or their legal representatives.

Patients were allocated to three study groups: sepsis group (*n* = 53), operated controls (*n* = 50), and nonoperated controls (*n* = 50). Inclusion criteria for the sepsis group were admission to SICU for the treatment of sepsis, sepsis presumed to be abdominal in origin, and at least one major abdominal surgical procedure performed during the current hospitalization. Inclusion criteria for the operated control group were American Society of Anesthesiologists (ASA) physical status 2, routine admission to SICU after major abdominal surgery, and uneventful recovery. Inclusion criteria for the nonoperated control group were ASA physical status 2, admission to the Department of Surgery prior to the major abdominal surgery, and no signs of inflammation or infection at the time of blood sampling. Major abdominal surgery was defined as a surgical procedure in which the peritoneum is entered, organs are removed, or normal anatomy is altered. Exclusion criteria were age < 18 years, sepsis of fungal or viral origin, sepsis of extra abdominal origin, chemotherapy and radiotherapy in the past 30 days, immunosuppressant therapy, major trauma, burns, end-stage organ disease, cardiogenic or hemorrhagic shock, and participation in another clinical trial during this hospitalization. All patients received routine treatment and care.

### 2.2. Clinical Assessment

Demographic (age, gender) and clinical (comorbidities, type of surgery, use of vasopressors/inotropes, use of mechanical ventilation, and 28-day survival) data were obtained from medical records. Sepsis was diagnosed according to the Surviving Sepsis Campaign criteria [25]. Assessment of comorbidities was performed in all patients using ASA physical status [26]. In septic patients, only comorbidities present prior to sepsis were considered and all the changes in physical status subsequently caused by sepsis were disregarded. In both control groups, ASA physical status was assessed prior to surgery. Disease severity scores, Acute Physiology and Chronic Health Evaluation II (APACHE II) score [27] and Sequential Organ Failure Assessment (SOFA) score [28], were calculated daily at the time of sample collection.

Sepsis-associated organ dysfunction was assessed using the following biomarker groups: inflammation—white blood cell (WBC) count, C-reactive protein (CRP), and procalcitonin (PCT); kidney injury—serum urea, creatinine, serum and urine neutrophil gelatinase-associated lipocalin (NGAL), and urine kidney injury molecule-1 (KIM-1); liver injury—aspartate aminotransferase (AST), alanine aminotransferase (ALT), and total and direct bilirubin; coagulation—platelet count, international normalized ratio (INR), and activated partial thromboplastin time (aPTT); and metabolic disorders—serum protein, glucose, and lactate. Use of vasopressors/inotropes was suggestive of cardiovascular dysfunction. Use of mechanical ventilation implied lung injury.

### 2.3. Blood and Urine Sample Collection

Blood and urine samples from the septic patients were collected during the first hour following admission to SICU (0 hour) and thereafter at 24, 48, 72, and 96 hours. Samples from the operated controls were collected once during the routine postoperative SICU stay and once from the nonoperated controls after fasting for the night.

### 2.4. MMP-9 and TIMP-1 Measurement and MMP-9/TIMP-1 Ratio Calculation

Blood was drawn into standard collection tubes with a cloth activator. Urine samples were collected using a standard catheter stream urine collection technique. Centrifuged serum and urine aliquots were then stored to −80°C until further analysis which was performed in batches. MMP-9 and TIMP-1 levels were measured using enzyme-linked immunosorbent assays (ELISA) (R&D Systems, MN, USA). Intra-assay precision (CV%) was 2.9% and 4.2% for MMP-9 and TIMP-1, respectively. Interassay precision (CV%) was 6.9% and 4.9% for MMP-9 and TIMP-1, respectively. The MMP-9/TIMP-1 ratio was calculated as follows: MMP-9/TIMP-1 ratio = MMP-9 (ng/mL)/TIMP-1 (ng/mL).

### 2.5. Other Laboratory Parameters

Blood samples were processed immediately after collection. CRP and PCT levels were measured using immunoturbidimetric and enzyme-linked fluorescent assays, respectively (bioMérieux, Lion, France). Serum and urine NGAL and urine KIM-1 levels were determined employing the ELISA technique (Abbott Diagnostics, IL, USA, and R&D Systems Inc., MN, USA, resp.). Serum urea, creatinine, AST, ALT, total and direct bilirubin, INR, aPTT, total protein, and glucose levels were measured using routine laboratory methods (Roche Diagnostics reagents using Roche Cobas c501, Mannheim, Germany). WBC count and platelet count were measured employing an automated hematology analyzer (ABX Horiba, Pentra DX 120, Montpellier, France). Lactate levels were measured using an automated blood gas analyzer (GEM Premier 3000, Instrumentation Laboratory, Milan, Italy).

### 2.6. Statistical Analysis

Statistical analysis was performed in SPSS Version 25 software (SPSS Inc., Chicago, IL, USA). Normality of the data distribution was evaluated by the Kolmogorov-Smirnov test. Categorical variables were reported as frequencies and continuous variables as medians and interquartile ranges. The Mann–Whitney *U* or chi-square test was used for comparisons between two independent samples and Friedman's repeated measures test for comparison between related samples. The diagnostic power of the tested biomarkers was evaluated by calculating the areas under receiver operating characteristic curves (AUC-ROC). Kaplan-Meier estimates of 28-day survival with the log-rank test were also performed. Correlations were analyzed employing the Kendall tau-b test. Both correlation and Kaplan-Meier analysis were performed using baseline values of the tested biomarkers as independent variables. The difference between the groups of septic patients over time was assessed using linear mixed models with compound symmetry as the repeated covariance type. The minimal statistical significance was set at two-tailed *p* < 0.05.

## 3. Results

### 3.1. Comparison between Patients with Sepsis and Control Groups

Baseline characteristics of the three patient groups are displayed in Table 1. Patient groups were similar in age, gender, and comorbidities represented by ASA scores. Septic patients and operated controls were similar in terms of the type of surgery. Patients with sepsis had higher disease severity scores compared to both control groups as did operated controls compared to nonoperated controls. On admission to SICU, septic patients had, compared to controls, lower MMP-9, MMP-9/TIMP-1 ratio, and protein levels but higher TIMP-1, WBC, CRP, urea, creatinine, NGAL, KIM-1, INR, aPTT, and blood glucose.

**Figure 1:**
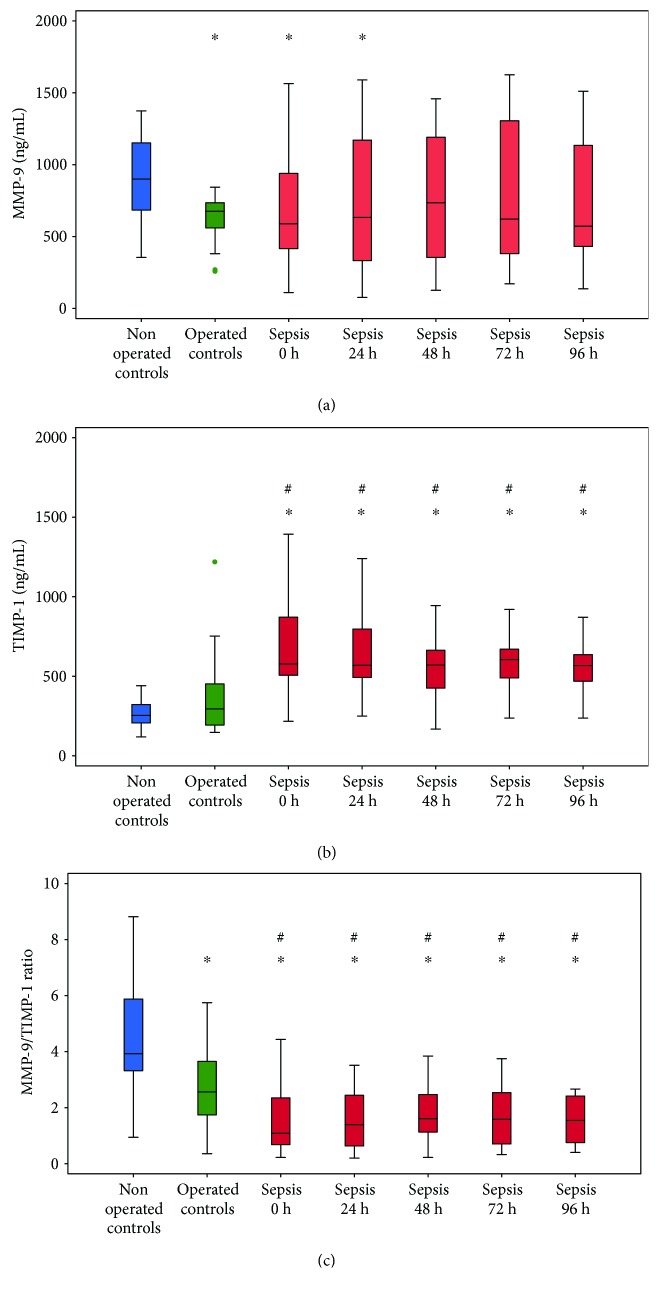
Dynamics of (a) MMP-9, (b) TIMP-1, and (c) MMP-9/TIMP-1 ratio in patients with sepsis in comparison to control groups. Data are expressed as minimum, maximum, median, and interquartile range. Blue—nonoperated controls, green—operated controls, and red—sepsis. ^∗^Statistically significant difference compared to nonoperated controls. ^#^Statistically significant difference compared to operated controls. Mann–Whitney *U* test; *p* < 0.05 is considered statistically significant. MMP-9: matrix metalloproteinase-9; TIMP-1: tissue inhibitor of matrix metalloproteinase-1.

Figure 1 depicts the dynamics of MMP-9, TIMP-1, and MMP-9/TIMP-1 ratio in patients with sepsis compared to both control groups. Levels of these biomarkers did not change significantly during 96 h following admission to SICU (*p* > 0.05 for all the three parameters). Patients with sepsis had lower levels of MMP-9 on admission to SICU and 24 h later than nonoperated controls, but such levels of MMP-9 were similar to those in operated controls (Figure 1(a)). Patients with sepsis had higher TIMP-1 levels (Figure 1(b)) and lower MMP-9/TIMP-1 ratios (Figure 1(c)) than both control groups at all time points. Operated controls also had lower MMP-9/TIMP-1 ratio relative to nonoperated controls (Figure 1(c)).

### 3.2. MMP-9, TIMP-1, and MMP-9/TIMP-1 Ratio as Diagnostic Biomarkers of Sepsis

The evaluation of MMP-9, TIMP-1, and MMP-9/TIMP-1 ratio as diagnostic biomarkers of sepsis was performed by the calculation of AUC-ROC values (Table 2). TIMP-1 and MMP-9/TIMP ratio were, unlike MMP-9, very good discriminators between patients with sepsis and both control groups at all time points. Also, MMP-9 and MMP-9/TIMP-1 ratio discriminated operated from nonoperated controls.

### 3.3. MMP-9, TIMP-1, and MMP-9/TIMP-1 Ratio as Prognostic Biomarkers of Sepsis

**Figure 2:**
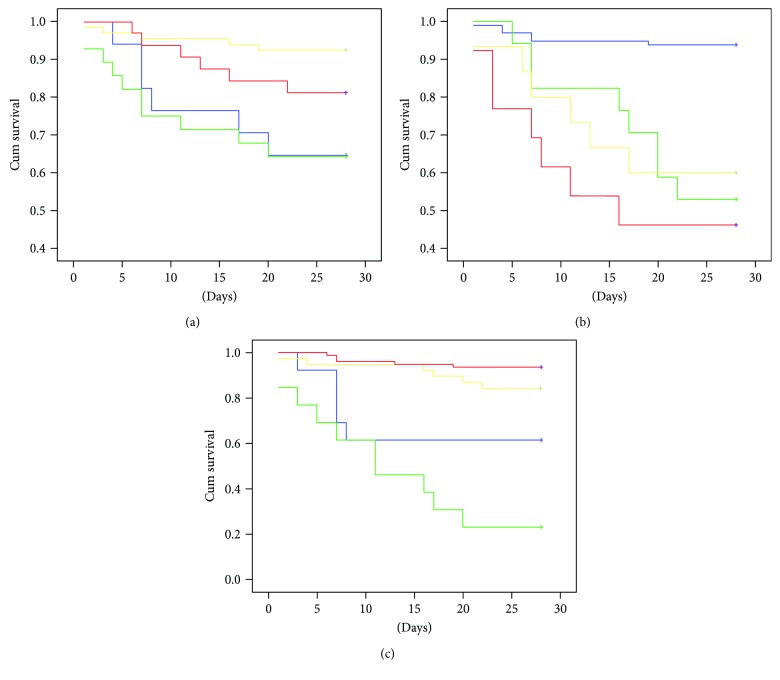
Kaplan-Meier estimates of 28-day survival based on (a) MMP-9, (b) TIMP-1, and (c) MMP-9/TIMP-1 ratio quartiles. Blue line—1st quartile, green line—2nd quartile, yellow line—3rd quartile, and red line—4th quartile. Log rank: (a) MMP-9 (*p* = 0.002), (b) TIMP-1 (*p* ≤ 0.001, and (c) MMP-9/TIMP-1 ratio (*p* < 0.001).

Twenty-eight septic patients (52.8%) died during the SICU stay. Lower MMP-9 (Figure 2(a)) and MMP-9/TIMP-1 ratio (Figure 2(c)) as well higher TIMP-1 (Figure 2(b)) were associated with shorter 28-day survival.

### 3.4. The Relationship between MMP-9, TIMP-1, and MMP-9/TIMP-1 Ratio and Sepsis-Associated Organ Dysfunction

Not surprisingly, MMP-9 and MMP-9/TIMP-1 ratio correlated directly with WBC count but indirectly with PCT levels. Lower MMP-9 and MMP-9/TIMP-1 ratio and higher TIMP-1 levels were associated with kidney and liver injury, coagulopathy, elevated lactate and glucose levels, and lower protein levels. MMP-9 and MMP-9/TIMP-1 ratio correlated negatively and TIMP-1 positively with disease severity scores (Table 3).

**Figure 3:**
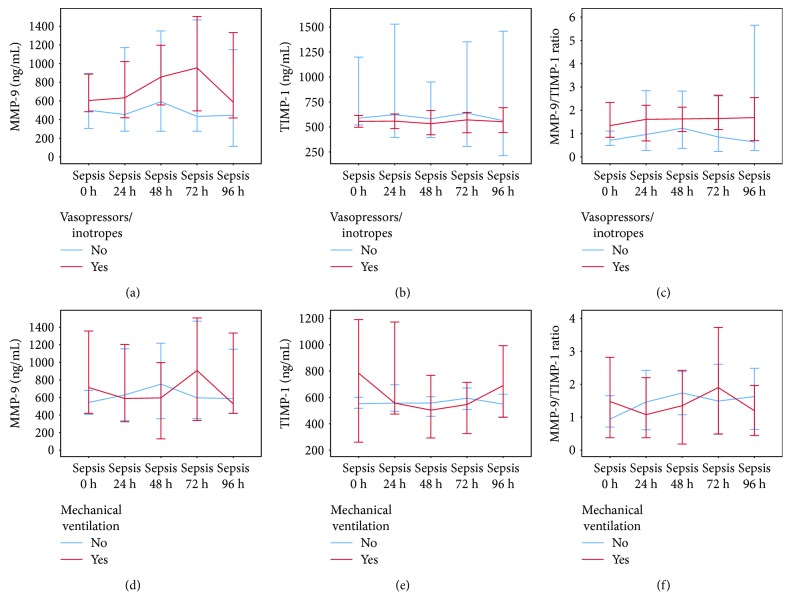
MMP-9, TIMP-1, and MMP-9/TIMP-1 ratio in septic patients receiving (a, b, c) vasopressors/inotropes or (d, e, f) mechanical ventilation. Data are presented as median and 95% CI. Linear mixed models—vasopressors/inotropes as a fixed factor: (a) *p*_MMP-9_ = 0.291, (b) *p*_TIMP-1_ = 0.026, and (c) *p*_MMP-9/TIMP-1_ = 0.316; mechanical ventilation as a fixed factor: (c) *p*_MMP-9_ = 0.715, (e) *p*_TIMP-1_ = 0.688, and (f) *p*_MMP-9/TIMP-1_ = 0.871. MMP-9: matrix metalloproteinase-9; TIMP-1: tissue inhibitor of matrix metalloproteinase-1.

Twenty-three (43.4%) septic patients were treated with vasopressors/inotropes, and 41 (77.4%) received mechanical ventilation. During 96 h following admission to SICU, TIMP-1 values were, unlike those of MMP-9 and MMP-9/TIMP-1 ratio, significantly higher in the septic patients treated with vasopressors/inotropes. Septic patients receiving mechanical ventilation had similar values of these biomarkers over time compared to the septic patients who did not receive mechanical ventilation (Figure 3).

## 4. Discussion

The principal novelties of this study are (1) the exploration of MMP-9, TIMP-1, and MMP-9/TIMP-1 ratio as biomarkers of sepsis specifically after major abdominal surgery and (2) the investigation of the potential relationships between these biomarkers and the sepsis-associated organ dysfunction.

### 4.1. Comparison between Patients with Sepsis and Control Groups

In our study, MMP-9 levels in septic patients were similar to those in operated controls but lower than those in nonoperated controls. According to literature, when septic patients were compared to healthy controls [10–13], higher MMP-9 levels were reported, but when compared to nonseptic ICU patients [9] or febrile controls [7], lower MMP-9 levels were found. It becomes clear that the study design has an important role in the interpretation of these results especially considering the influence of comorbidities [5, 6] and major surgery [27, 28] on MMP-9 levels. The unique design of our study enabled control over the influence of these confounding factors. Our results also suggest that major abdominal surgery, independent of sepsis, is associated with lower MMP-9 levels. Additionally, there is evidence of postoperative MMP-9 storage depletion [29] and lowering of MMP-9 levels related to fluid administration during major surgery [30].

As opposed to MMP-9, TIMP-1 levels are repeatedly reported to be higher in sepsis [7, 8, 10–13]. Not surprisingly, our patients also had higher TIMP-1 levels and, consequently, lower MMP-9/TIMP-1 ratios compared to both control groups at all time points.

### 4.2. MMP-9, TIMP-1, and MMP-9/TIMP-1 Ratio as Diagnostic Biomarkers of Sepsis

ROC curve analysis showed that TIMP-1 and MMP-9/TIMP-1 ratio were very good diagnostic biomarkers of sepsis after major abdominal surgery. Furthermore, AUC-ROC values were similar at all time points which suggests that their diagnostic value remains relevant for at least 96 h. This feature could be potentially very useful if recognition of sepsis is delayed which, unfortunately, is not uncommon in daily clinical practice [25]. AUC-ROC values were quite high and comparable to those in pediatric sepsis [7] and were even slightly higher than those of PCT, the established biomarker of sepsis [31, 32]. Our results also showed that MMP-9 was a poor discriminator between the septic patients and controls. Martin et al. found that MMP-9 had only moderate capacity to diagnose sepsis (AUC-ROC 0.73 (0.65–0.80)) [9]. Poor diagnostic performance of MMP-9 is not surprising considering the large variability of the reported values [7–10, 13].

### 4.3. MMP-9, TIMP-1, and MMP-9/TIMP-1 Ratio as Prognostic Biomarkers of Sepsis

Our results suggest that lower MMP-9 and MMP-9/TIMP-1 ratio but higher TIMP-1 levels are associated with higher mortality rates and, accordingly, higher disease severity scores. Previous studies also demonstrated lower MMP-9 [8, 9, 15] and higher TIMP-1 [8, 12, 13, 15] in nonsurvivors and in patients with higher disease severity scores [6, 12]. Lorente et al. [8] and Wang et al. [13], as opposed to Martin et al. [9] and Serrano-Gomez et al. [14], supported our findings that MMP-9, TIMP-1, and MMP-9/TIMP-1 ratio are good prognostic biomarkers of 28-day survival. A recent study by Niño et al., however, demonstrated that only TIMP-1 and not MMP-9 and MMP-9/TIMP-1 ratio was a good predictor of mortality in sepsis [15].

Previously discussed diagnostic and prognostic value of the proposed biomarkers suggests that they should be explored in larger patient populations. Considering that these biomarkers were not used in routine clinical practice, it is difficult to estimate their economic impact. However, since the established biomarkers of sepsis, in particular PCT and CRP, are costly assayed, there is an opportunity for more affordable ones to gain clinical acceptance. On that note, given that MMP-9 is inferior in its diagnostic and prognostic performance to TIMP-1, the use of the MMP-9/TIMP-1 ratio could not be economically justified.

### 4.4. The Relation between MMP-9, TIMP-1, and MMP-9/TIMP-1 Ratio and Sepsis-Associated Organ Dysfunction

WBC count, CRP, and PCT are widely used in clinical practice as diagnostic and prognostic biomarkers of inflammation, infection, and sepsis [33, 34]. Our study reports a positive correlation between WBC count and MMP-9 levels that was expected considering the origin of MMP-9 [6]. Similar to earlier reports [7, 9], MMP-9, TIMP-1, and MMP-9/TIMP-1 ratio were not significantly related to CRP levels. Wang et al. found a positive correlation between PCT and TIMP-1 [13] while our results suggest a negative correlation between PCT and MMP-9. A negative correlation between PCT and MMP-9 is expected given the association of higher PCT levels with more severe forms of sepsis [25, 33]. According to our results, lower MMP-9 levels could be found in patients with higher disease severity scores.

In our study, low MMP-9 and high TIMP-1 levels were associated with more severe kidney and liver injury. This was supported by Renckens et al. who demonstrated that MMP-9-knockout mice with abdominal sepsis developed more severe distant organ damage [21]. On the other hand, elevated MMP-9 and TIMP-1 expression was found in kidneys and livers of animal models of sepsis [18, 21]. A more detailed comment on the role of these biomarkers in septic AKI could be found in our earlier paper [17], but clinical reports on MMP-9 and TIMP-1 in sepsis-associated liver injury are lacking.

The association of MMP-9 and TIMP-1 with platelet count, INR, and aPTT is not surprising given the previous findings of Lorente et al. [8] and the more complex coagulation-independent interaction between platelets and MMP-9 [35].

According to our results, low MMP-9 and MMP-9/TIMP-1 ratio but high TIMP-1 values were associated with lower protein and higher blood glucose and lactate levels which is consistent with some previous finding [8, 36].

We found that TIMP-1 values were significantly higher in the septic patients treated with vasopressors/inotropes. Clinical studies on this topic are lacking, but experimental models imply that catecholamines have a very complex interaction with MMPs and their inhibitors [37, 38] that could be influenced by various medications, none of which are accounted for in this research.

Septic patients receiving mechanical ventilation had similar MMP-9 and TIMP-1 values compared to the septic patients who did not receive mechanical ventilation. Possible explanation could be found in the interaction of the opposing factors influencing the levels of these biomarkers. Sepsis-associated lung injury is linked to elevated MMP-9 and TIMP-1 lung expression [18–21], but mechanical ventilation per se is related to reduction in MMP-9 and TIMP-1 levels [39]. Also, beta-agonists have a potential to alter MMP-9 and TIMP-1 levels [40]. Additionally, the use of mechanical ventilation could be prompted by reasons other than lung injury (hemodynamic instability, increase in intracranial pressure, etc.).

Our study has several limitations. Firstly, it is possible that the relatively small number of patients could have added to the large variability in the data. Secondly, due to the limit to the number of samples which could be processed, samples from the control groups were taken only once instead of five times. Thirdly, due to a limited budget, CRP, PCT, and lactate levels were not measured in all patients. Finally, aside from age, gender, surgery, and comorbidities, we did not take into account any other possible confounding factors such as the use of corticosteroids, certain antibiotics, and amount of fluids received.

## 5. Conclusions

TIMP-1, unlike MMP-9 and MMP-9/TIMP-1 ratio, was a good diagnostic and prognostic biomarker of sepsis after major abdominal surgery. These biomarkers were also associated with inflammation, sepsis-associated kidney and liver injury, coagulopathy, and metabolic disorders. Future studies could possibly provide more insight into MMP-9 and TIMP-1's role in the pathophysiology of the sepsis-associated organ dysfunction.

## Figures and Tables

**Table 1:** Baseline demographic, clinical, and laboratory characteristics.

	Nonoperated controls (*N* = 50)	Operated controls (*N* = 50)	Sepsis (*N* = 53)
*Demographic and clinical characteristics*			
Age (years)	60.00 (54.75–72.00)	65.00 (57.75–74.00)	70.00 (60.00–75.50)
Gender (m/f)	23/27	22/28	28/25
ASA (1/2/3/4/5)	0/50/0/0/0	0/50/0/0/0	2/47/4/0/0
Surgery (upper gastrointestinal/colorectal)	NA	9/41	18/35
*Disease severity scores*			
APACHE II	4.00 (2.00–5.25)	7.00 (5.00–7.50)^a^	21.50 (16.00–28.00)^b,c^
SOFA	0 (0–0)	0 (0–0)	7 (4–10)^b,c^
*Biomarkers of inflammation*			
MMP-9 (ng/mL)	895.6 (665.5–1157.9)	663.3 (537.2–726.1)^a^	572.9 (391.5–997.8)^b^
TIMP-1 (ng/mL)	219.9 (170.6–292.4)	263.0 (153.2–428.0)	558.7 (469.9–875.8)^b,c^
MMP-9/TIMP-1 ratio	3.88 (3.26–5.93)	2.47 (1.59–3.61)^a^	0.94 (0.50–2.27)^b,c^
WBC count × 10^9^/L	7.0 (5.5–8.2)	9.1 (6.5–12.1)^a^	13.8 (9.0–18.6)^b,c^
CRP (mg/L)	—	97.5 (14.8–157.2)	197.8 (148.3–256.1)^c^
PCT (*μ*g/L)	—	—	12.96 (3.17–30.47)
*Biomarkers of kidney injury*			
Urea (mmol/L)	4.8 (3.7–5.7)	4.9 (3.6–6.7)	12.9 (8.2–19.7)^b,c^
Creatinine (*μ*mol/L)	79.0 (65.0–85.0)	74.0 (56.8–92.0)	133.3 (93.5–203.2)^b,c^
Serum NGAL (ng/mL)	87.3 (82.2–95.3)	100.3 (91.2–111.9)^a^	169.0 (105.5–200.9)^b,c^
Urine NGAL (ng/mL)	14.6 (7.8–68.9)	42.9 (27.2–57.6)^a^	107.1 (46.7–204.5)^b,c^
Urine KIM-1 (pg/mL)	740.5 (233.5–919.3)	800.0 (730.0–1977.0)^a^	790.0 (712.0–1309.7)^b^
*Biomarkers of liver injury*			
AST (IU/L)	19 (16–25)	29 (19–111)^a^	47 (28–118)^b^
ALT (IU/L)	21 (14–34)	31 (19–99)	35 (18–78)^b^
Total bilirubin (*μ*mol/L)	9.1 (5.9–18.8)	17.3 (10.1–36.2)^a^	16.8 (11.8–46.1)^b^
Direct bilirubin (*μ*mol/L)	3.30 (2.05–8.55)	8.85 (5.97–20.05)^a^	6.75 (5.40–26.42)^b^
*Biomarkers of coagulation*			
Platelets × 10^9^/L	254 (199–291)	198 (177–265)^a^	223 (142–316)
INR	1.21 (1.03–1.38)	1.19 (1.05–1.32)	1.41 (1.13–1.96)^b,c^
aPTT (s)	35 (28–41)	36 (27–43)	46 (37–59)^b,c^
*Biomarkers of metabolic disorders*			
Protein (g/L)	66.9 (61.8–73.0)	56.5 (45.8–62.2)^a^	35.1 (28.5–45.9)^b,c^
Glucose (mmol/L)	5.6 (5.0–6.1)	6.4 (5.9–8.3)^a^	8.2 (5.8–11.1)^b,c^
Lactate (mmol/L)	—	—	1.30 (1.00–3.90)

Data are presented as median and 25th–75th percentile values or frequencies. ^a^Significant difference between operated and nonoperated controls, ^b^significant difference between the sepsis group and nonoperated controls, and ^c^significant difference between the sepsis group and operated controls. Mann–Whitney *U* or chi-square test; *p* < 0.05 is considered statistically significant. ASA: American Society of Anesthesiologists physical status; APACHE II: Acute Physiology and Chronic Health Evaluation II score; SOFA: Sequential Organ Failure Assessment score; MMP-9: matrix metalloproteinase-9; TIMP-1: tissue inhibitor of matrix metalloproteinase-1; WBC: white blood cell; CRP: C-reactive protein; PCT: procalcitonin; NGAL: neutrophil gelatinase-associated lipocalin; KIM-1: kidney injury molecule-1; AST: aspartate aminotransferase; ALT: alanine aminotransferase; INR: international normalized ratio; aPTT: activated partial thromboplastin time.

**Table 2:** MMP-9, TIMP-1, and MMP-9/TIMP-1 ratio as diagnostic biomarkers of sepsis.

	AUC-ROC (95% CI)		
	MMP-9	TIMP-1	MMP-9/TIMP-1 ratio
*Nonoperated controls versus operated controls*			
0 h	0.798 (0.702–0.895)^∗^	0.584 (0.465–0.704)	0.761 (0.664–0.858)^∗^
*Sepsis* versus *nonoperated controls*			
0 h	0.675 (0.561–0.789)^∗^	0.940 (0.889–0.991)^∗^	0.924 (0.873–0.975)^∗^
24 h	0.640 (0.511–0.769)^∗^	0.972 (0.941–1.004)^∗^	0.929 (0.878–0.980)^∗^
48 h	0.593 (0.450–0.736)	0.929 (0.862–0.997)^∗^	0.914 (0.853–0.975)^∗^
72 h	0.591 (0.417–0.765)	0.951 (0.892–1.006)^∗^	0.901 (0.821–0.980)^∗^
96 h	0.646 (0.457–0.826)	0.925 (0.825–1.026)^∗^	0.911 (0.825–0.997)^∗^
*Sepsis versus operated controls*			
0 h	0.541 (0.418–0.664)	0.854 (0.778–0.930)^∗^	0.788 (0.697–0.879)^∗^
24 h	0.513 (0.372–0.653)	0.870 (0.797–0.943)^∗^	0.753 (0.650–0.855)^∗^
48 h	0.570 (0.415–0.724)	0.820 (0.728–0.913)^∗^	0.718 (0.605–0.832)^∗^
72 h	0.523 (0.342–0.703)	0.857 (0.768–0.913)^∗^	0.724 (0.593–0.854)^∗^
96 h	0.514 (0.307–0.721)	0.845 (0.738–0.952)^∗^	0.750 (0.612–0.888)^∗^

Data are presented as AUC-ROC (95% CI). ^∗^*p* < 0.05. AUC-ROC: area under receiver operating characteristic curve; MMP-9: matrix metalloproteinase-9; TIMP-1: tissue inhibitor of matrix metalloproteinase-1.

**Table 3:** Correlation analysis.

	MMP-9	TIMP-1	MMP-9/TIMP-1 ratio
*Disease severity scores*			
APACHE II	−0.099	0.107	−0.116^∗^
SOFA	−0.149^∗∗^	0.181^∗∗^	−0.196^∗∗^
*Biomarkers of inflammation*			
WBC	0.377^∗∗^	0.018	0.268^∗∗^
CRP	−0.047	0.019	−0.037
PCT	−0.246^∗∗^	0.150	−0.214^∗∗^
*Biomarkers of kidney injury*			
Urea	−0.154^∗∗^	0.103	−0.167^∗∗^
Creatinine	−0.187^∗∗^	0.153^∗∗^	−0.234^∗∗^
Serum NGAL	−0.089	0.271^∗∗^	−0.203^∗∗^
Urine NGAL	−0.277^∗∗^	0.197^∗∗^	−0.338^∗∗^
KIM-1	0.002	0.144^∗∗^	−0.104
*Biomarkers of liver injury*			
AST	−0.145^∗^	0.236^∗∗^	−0.210^∗∗^
ALT	−0.051	0.148^∗^	−0.097
Total bilirubin	−0.290^∗∗^	0.102	−0.266^∗∗^
Direct bilirubin	−0.299^∗∗^	0.143	−0.263^∗∗^
*Biomarkers of coagulation*			
Platelets	0.371^∗∗^	−0.103	0.329^∗∗^
INR	−0.112	0.153^∗^	−0.127
aPTT	−0.143^∗^	0.102^∗^	0.135^∗^
*Biomarkers of metabolic disorders*			
Total protein	0.113^∗^	−0.109^∗^	0.120^∗^
Glucose	−0.062	0.270^∗∗^	−0.246^∗∗^
Lactate	−0.211^∗∗^	0.263^∗∗^	−0.275^∗∗^

Kendall's Tau-b correlation analysis. ^∗∗^The correlation is significant at the 0.01 level (2-tailed); ^∗^ the correlation is significant at the 0.05 level (2-tailed). MMP-9: matrix metalloproteinase-9; TIMP-1: tissue inhibitor of matrix metalloproteinase-1; APACHE II: Acute Physiology and Chronic Health Evaluation II score; SOFA: Sequential Organ Failure Assessment score; WBC: white blood cell; CRP: C-reactive protein; PCT: procalcitonin; NGAL: neutrophil gelatinase-associated lipocalin; KIM-1: kidney injury molecule-1; AST: aspartate aminotransferase; ALT: alanine aminotransferase; INR: international normalized ratio; aPTT: activated partial thromboplastin time.

## Data Availability

The data used to support the findings of this study are available from the corresponding author upon request.

## References

[1] Hecker A., Uhle F., Schwandner T., Padberg W., Weigand M. A. (2014). Diagnostics, therapy and outcome prediction in abdominal sepsis: current standards and future perspectives.

[2] Wichmann M. W., Inthorn D., Andress H.-J., Schildberg F. W. (2000). Incidence and mortality of severe sepsis in surgical intensive care patients: the influence of patient gender on disease process and outcome.

[3] Dellinger R. P. (2013). The Surviving Sepsis Campaign: 2013 and beyond.

[4] Khokha R., Murthy A., Weiss A. (2013). Metalloproteinases and their natural inhibitors in inflammation and immunity.

[5] Galliera E., Tacchini L., Corsi Romanelli M. M. (2015). Matrix metalloproteinases as biomarkers of disease: updates and new insights.

[6] Vandooren J., Van den Steen P. E., Opdenakker G. (2013). Biochemistry and molecular biology of gelatinase B or matrix metalloproteinase-9 (MMP-9): the next decade.

[7] Alqahtani M. F., Smith C. M., Weiss S. L., Dawson S., Ralay Ranaivo H., Wainwright M. S. (2016). Evaluation of new diagnostic biomarkers in pediatric sepsis: matrix metalloproteinase-9, tissue inhibitor of metalloproteinase-1, mid-regional pro-atrial natriuretic peptide, and adipocyte fatty-acid binding protein.

[8] Lorente L., Martín M. M., Solé-Violán J. (2014). Association of sepsis-related mortality with early increase of TIMP-1/MMP-9 ratio.

[9] Martin G., Asensi V., Montes A. H. (2014). Role of plasma matrix-metalloproteases (MMPs) and their polymorphisms (SNPs) in sepsis development and outcome in ICU patients.

[10] Mühl D., Nagy B., Woth G. (2011). Dynamic changes of matrix metalloproteinases and their tissue inhibitors in severe sepsis.

[11] Yazdan-Ashoori P., Liaw P., Toltl L. (2011). Elevated plasma matrix metalloproteinases and their tissue inhibitors in patients with severe sepsis.

[12] Lauhio A., Hästbacka J., Pettilä V. (2011). Serum MMP-8, -9 and TIMP-1 in sepsis: high serum levels of MMP-8 and TIMP-1 are associated with fatal outcome in a multicentre, prospective cohort study. Hypothetical impact of tetracyclines.

[13] Wang M., Zhang Q., Zhao X., Dong G., Li C. (2014). Diagnostic and prognostic value of neutrophil gelatinase-associated lipocalin, matrix metalloproteinase-9, and tissue inhibitor of matrix metalloproteinases-1 for sepsis in the emergency department: an observational study.

[14] Serrano-Gomez S., Burgos-Angulo G., Niño-Vargas D. C. (2017). Predictive value of matrix metalloproteinases and their inhibitors for mortality in septic patients: a cohort study.

[15] Niño M. E., Serrano S. E., Niño D. C. (2017). TIMP1 and MMP9 are predictors of mortality in septic patients in the emergency department and intensive care unit unlike MMP9/TIMP1 ratio: multivariate model.

[16] White L. E., Chaudhary R., Moore L. J., Moore F. A., Hassoun H. T. (2011). Surgical sepsis and organ crosstalk: the role of the kidney.

[17] Bojic S., Kotur-Stevuljevic J., Kalezic N. (2015). Diagnostic value of matrix metalloproteinase-9 and tissue inhibitor of matrix metalloproteinase-1 in sepsis-associated acute kidney injury.

[18] Teng L., Yu M., Li J. M. (2012). Matrix metalloproteinase-9 as new biomarkers of severity in multiple organ dysfunction syndrome caused by trauma and infection.

[19] Lingaraju M. C., Pathak N. N., Begum J. (2015). Betulinic acid attenuates lung injury by modulation of inflammatory cytokine response in experimentally-induced polymicrobial sepsis in mice.

[20] Jin L. Y., Li C. F., Zhu G. F., Wu C. T., Wang J., Yan S. F. (2014). Effect of siRNA against NF-*κ*B on sepsis-induced acute lung injury in a mouse model.

[21] Renckens R., Roelofs J. J. T. H., Florquin S. (2006). Matrix metalloproteinase-9 deficiency impairs host defense against abdominal sepsis.

[22] Dal-Pizzol F., Rojas H. A., dos Santos E. M. (2013). Matrix metalloproteinase-2 and metalloproteinase-9 activities are associated with blood–brain barrier dysfunction in an animal model of severe sepsis.

[23] de la Torre E., Hovsepian E., Penas F. N. (2013). Macrophages derived from septic mice modulate nitric oxide synthase and angiogenic mediators in the heart.

[24] Cui N., Wang H., Long Y., Su L., Liu D. (2015). Dexamethasone suppressed LPS-induced matrix metalloproteinase and its effect on endothelial glycocalyx shedding.

[25] Dellinger R. P., Levy M. M., Rhodes A. (2013). Surviving Sepsis Campaign: international guidelines for management of severe sepsis and septic shock, 2012.

[26] Menke H., Klein A., John K. D., Junginger T. (1993). Predictive value of ASA classification for the assessment of the perioperative risk.

[27] Berger M. M., Marazzi A., Freeman J., Chiolero R. (1992). Evaluation of the consistency of Acute Physiology and Chronic Health Evaluation (APACHE II) scoring in a surgical intensive care unit.

[28] Vincent J.-L., Moreno R., Takala J. (1996). The SOFA (Sepsis-Related Organ Failure Assessment) score to describe organ dysfunction/failure. On behalf of the Working Group on Sepsis-Related Problems of the European Society of Intensive Care Medicine.

[29] Belizon A., Kirman I., Karten M., Jain S., Whelan R. L. (2005). Rapid increase in serum levels of matrix metalloproteinase-9 (MMP-9) postoperatively is associated with a decrease in the amount of intracellular MMP-9.

[30] Volta C. A., Trentini A., Farabegoli L. (2013). Effects of two different strategies of fluid administration on inflammatory mediators, plasma electrolytes and acid/base disorders in patients undergoing major abdominal surgery: a randomized double blind study.

[31] Wacker C., Prkno A., Brunkhorst F. M., Schlattmann P. (2013). Procalcitonin as a diagnostic marker for sepsis: a systematic review and meta-analysis.

[32] Enguix-Armada A., Escobar-Conesa R., Garcia-De La Torre A., de La Torre-Prados M. V. (2016). Usefulness of several biomarkers in the management of septic patients: C-reactive protein, procalcitonin, presepsin and mid-regional pro-adrenomedullin.

[33] Arora S., Singh P., Singh P. M., Trikha A. (2015). Procalcitonin levels in survivors and nonsurvivors of sepsis: systematic review and meta-analysis.

[34] Lelubre C., Anselin S., Zouaoui Boudjeltia K., Biston P., Piagnerelli M. (2013). Interpretation of C-reactive protein concentrations in critically ill patients.

[35] Rahman M., Zhang S., Chew M., Syk I., Jeppsson B., Thorlacius H. (2013). Platelet shedding of CD40L is regulated by matrix metalloproteinase-9 in abdominal sepsis.

[36] Sachwani G. R., Jaehne A. K., Jayaprakash N. (2016). The association between blood glucose levels and matrix-metalloproteinase-9 in early severe sepsis and septic shock.

[37] Yan A. T., Yan R. T., Spinale F. G. (2008). Relationships between plasma levels of matrix metalloproteinases and neurohormonal profile in patients with heart failure.

[38] Yin X., Zhou L., Han F. (2017). Beta-adrenoceptor activation by norepinephrine enhances lipopolysaccharide-induced matrix metalloproteinase-9 expression through the ERK/JNK-c-Fos pathway in human THP-1 cells.

[39] Beer L., Warszawska J. M., Schenk P. (2015). Intraoperative ventilation strategy during cardiopulmonary bypass attenuates the release of matrix metalloproteinases and improves oxygenation.

[40] O’Kane C. M., McKeown S. W., Perkins G. D. (2009). Salbutamol up-regulates matrix metalloproteinase-9 in the alveolar space in the acute respiratory distress syndrome.

